# Preferential Response of Basal-Like Head and Neck Squamous Cell Carcinoma Cell Lines to EGFR-Targeted Therapy Depending on *EREG*-Driven Oncogenic Addiction

**DOI:** 10.3390/cancers11060795

**Published:** 2019-06-08

**Authors:** Sylvie Job, Aurélien de Reyniès, Betty Heller, Amélie Weiss, Eric Guérin, Christine Macabre, Sonia Ledrappier, Cyril Bour, Christine Wasylyk, Nelly Etienne-Selloum, Laurent Brino, Christian Gaiddon, Bohdan Wasylyk, Alain C. Jung

**Affiliations:** 1 Programme Cartes d’Identité des Tumeurs (CIT), Ligue Nationale Contre le Cancer, 75013 Paris, France; Sylvie.Job@ligue-cancer.net (S.J.); Aurelien.DeReynies@ligue-cancer.net (A.d.R.); 2 Institut de Génétique et de Biologie Moléculaire et Cellulaire IGBMC, UMR 7104 CNRS-UdS, U.1258 INSERM, 1 rue Laurent Fries, BP 10142, 67404 Illkirch CEDEX, France; heller@igbmc.fr (B.H.); weiss@igbmc.fr (A.W.); christannie67@gmail.com (C.W.); brino@igbmc.fr (L.B.); boh@igbmc.fr (B.W.); 3 Laboratoire de Biochimie et Biologie Moléculaire, Hôpitaux Universitaires de Strasbourg, 67098 Strasbourg, France; eric.guerin@chru-strasbourg.fr; 4 Université de Strasbourg, Inserm, UMR_S1113, 67200 Strasbourg, France; cmacabre@strasbourg.unicancer.fr (C.M.); sledrappier@strasbourg.unicancer.fr (S.L.); cbour@strasbourg.unicancer.fr (C.B.); gaiddon@unistra.fr (C.G.); 5 Centre de Lutte Contre le Cancer Paul Strauss, 67000 Strasbourg, France; nelly.etienne-selloum@unistra.fr; 6 UMR 7021 CNRS/Unistra, Laboratoire de Bioimagerie et Pathologies (LBP), Faculté de Pharmacie, 67401 Illkirch, France

**Keywords:** head and neck squamous cell carcinoma, cancer subgroups, EGFR, EREG, drug sensitivity, treatment combinations

## Abstract

The management of locally advanced head and neck squamous cell carcinoma (HNSCC) with Cetuximab, a monoclonal antibody targeting the epidermal growth factor receptor (EGFR), achieves only moderate response rates, and clinical trials that evaluated EGFR-blockade with tyrosine kinase inhibitors (TKI) yielded disappointing results. Inter-tumor heterogeneity may hinder the therapeutic efficiency of anti-EGFR treatments. HNSCC heterogeneity was addressed in several studies, which all converged towards the definition of molecular subgroups. They include the basal subgroup, defined by the deregulated expression of factors involved in the EGFR signaling pathway, including the epiregulin EGFR ligand encoded by the *EREG* gene. These observations indicate that basal tumors could be more sensitive to anti-EGFR treatments. To test this hypothesis, we performed a screen of a representative collection of basal versus non-basal HNSCC cell lines for their sensitivity to several anti-EGFR drugs (Cetuximab, Afatinib, and Gefitinib), tested as monotherapy or in combination with drugs that target closely-linked pathways [Mitogen-activated protein kinase kinase/extracellular signal–regulated kinases (MEK), mammalian Target of Rapamycine (mTOR) or Human Epidermal growth factor Receptor 2 (HER2)]. Basal-like cell lines were found to be more sensitive to EGFR blockade alone or in combination with treatments that target MEK, mTOR, or HER2. Strikingly, the basal-like status was found to be a better predictor of cell response to EGFR blockade than clinically relevant mutations [e.g., cyclin-dependent kinase Inhibitor 2A (*CDKN2A*)]. Interestingly, we show that EGFR blockade inhibits *EREG* expression, and that *EREG* knock-down decreases basal cell clonogenic survival, suggesting that *EREG* expression could be a predictive functional marker of sensitivity to EGFR blockade in basal-like HNSCC.

## 1. Introduction

Head and neck squamous cell carcinoma (HNSCC) is the sixth most common cancer in the world, with over 600,000 cases diagnosed each year [1]. They encompass a heterogeneous group of malignancies that arise from the epithelium of the upper aero-digestive tract (oral cavity, pharynx, and larynx). The major risk factors are heavy consumption of tobacco smoke and alcohol, and infection with human papillomaviruses (HPV) [2]. Despite aggressive multi-modal treatment modalities (involving surgery, radiotherapy, and chemotherapy), recurrence occurs in about 50% of patients, mainly locoregionally, but also locally and at distance (see [3] and references therein). The prognosis of locally advanced HNSCC is poor, with <50% of the patients remaining alive five years after treatment [1]. Cetuximab (a monoclonal antibody that targets EGFR (epidermal growth factor receptor)) is the only targeted therapy used for the management of locally advanced HNSCC. However, more than 10 years after the Food and Drug Administration approval of Cetuximab, alone or in combination with radiotherapy or chemotherapy, HNSCC patient outcome has only been moderately improved [4].

Several “omic” analyses have established that HNSCC is molecularly heterogeneous. A seminal study by Chung et al. stratified HNSCC into four distinct gene-expression subtypes [5]. Group 1 displays EGFR-pathway and hypoxia-related molecular signatures. Group 2 is mesenchymal-marker enriched, consistent with the presence of fibroblasts and having undergone an epithelial-to-mesenchymal transition. Group 3 has features of normal epithelia. Group 4 expresses high levels of transcripts coding for antioxidant and detoxification enzymes, which possibly reflects exposure to tobacco smoke. Walter et al. [6] identified four gene-expression subgroups related to Groups 1–4, that they named basal, mesenchymal, atypical, and classical, respectively. The Cancer Genome Atlas consortium [7] refined this classification, using a comprehensive multi-platform analysis of copy number alterations, somatic mutations, gene expression variations and DNA methylation profiles of 279 tumor samples. Keck et al. [8] described five subgroups, three HPV-negative (basal, classical non-HPV, and mesenchymal non-HPV) and two HPV-positive (classical HPV and mesenchymal HPV) HNSCCs. Finally, Cecco et al. [9] reported six subtypes (immunoreactive, inflammatory, HPV-like, classical, hypoxia-associated, and mesenchymal), using a meta-analysis of online gene-expression data-sets. However, the clinical relevance of these subtypes is uncertain, given the reported discrepancies between molecular subtypes and outcome [5,6,8].

Here, we report that there are common molecular features that define four subtypes, including a basal subtype. There are some indications that the basal subtype is particularly sensitive to EGFR blockade, but there is a lack of consistent studies. Patients with basal HNSCC have been shown to respond better to platinum-based chemotherapy combined with Cetuximab [10]. The Genomics Drug Sensitivity in Cancer project provides some data about the in vitro cell response to Gefitinib [11]. However, these studies are hardly comparable, since the anti-EGFR drugs were not tested on the same set of cell lines. In order to evaluate the efficiency of several anti-EGFR drugs (including Cetuximab) on the same set of samples, we designed an in vitro screen of 25 well-characterized HNSCC cell lines that were classified as basal or non-basal. Three clinically-used EGFR inhibitors (Cetuximab, Erlotinib, and Afatinib) were tested as monotherapies. Given the resistance that eventually occurs after targeted EGFR blockade [12], we also tested these three anti-EGFR drugs in combination with inhibitors of downstream pathways (mTOR/Phosphatidylinositol 3-kinase (PI3K) and MEK) or another tyrosine kinase receptor of the avian erythroblastosis oncogene B (ErbB)/HER family (HER2). We showed that basal-like cells are more sensitive to EGFR blockade with EGFR inhibitors alone and in drug combinations.

Basal-like HNSCC aberrantly expresses factors involved in EGFR signaling, including the up-regulation of the EGFR ligand epiregulin. Epiregulin, which is encoded by the *EREG* gene, is expressed as a type I transmembrane precursor, and extracellular domain cleavage leads to autocrine and/or paracrine activation of EGFR and ErbB4/HER4 via the release of mature, active ligands [13]. Epiregulin appears to have a particularly important role in several human cancers by regulating cell proliferation and migration [14]. Interestingly, *EREG* overexpression is thought to fuel an oncogenic feedback loop that activates signaling pathways downstream of EGFR/ErbB4 and was proposed to be a therapeutic target in non-small-cell lung carcinoma [15]. Epiregulin expression has also been shown to be a predictive biomarker of response to anti-EGFR therapies in metastatic colorectal cancer [16]. Intriguingly, Cecco et al. and Bossi et al. suggested that HNSCC patients with tumors of the basal subgroup would be more sensitive to treatments targeting EGFR [9,10]. We show that EGFR blockade preferentially inhibits *EREG* expression in basal-like cells, and that direct inhibition of *EREG* with siRNAs inhibits cell survival. These results support the hypothesis that high *EREG* expression could be a predictive functional marker of sensitivity to EGFR blockade in basal-like HNSCC.

## 2. Results

### 2.1. A Common Molecular Basal-Like Subgroup Can Be Distinguished in Different HNSCC Data Sets

**Figure 1:**
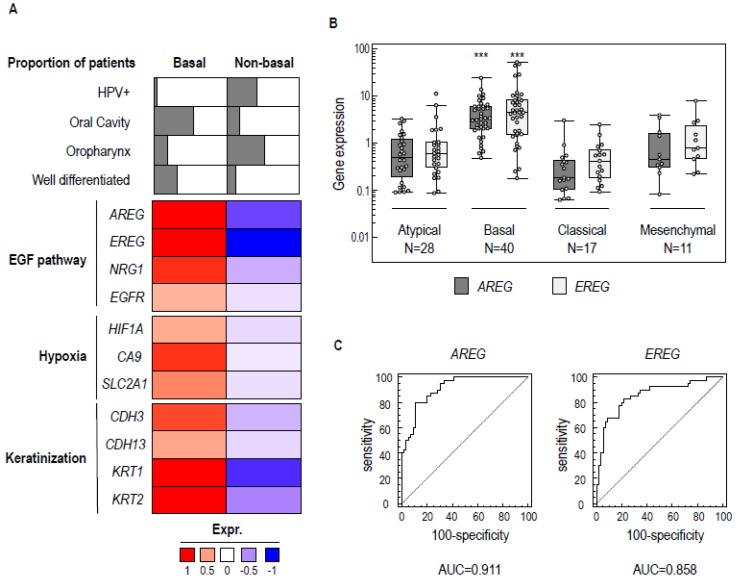
Molecular features of basal head and neck squamous cell carcinoma (HNSCC). (**A**) Summary of the main pathology features and pathway enrichment analysis of the basal HNSCC subgroup in public omic data sets [5,6,7,8]. (**B**) Boxplot of the gene expression of *AREG* and *EREG* genes measured by RT-qPCR in basal, mesenchymal, atypical, and classical HNSCC. Expression levels were compared between tumor subgroups and were found to be significantly higher in basal HNSCC compared to other molecular subgroups (ANOVA *** *p* < 0.001). (**C**) Receiver Operating Characteristic (ROC) curve analyses of the ability of *AREG* and *EREG* gene expression levels to discriminate between basal and non-basal HNSCC. The area under the curve (AUC), corresponding to the optimal specificity and sensitivity, is shown.

Over the last decade, several molecular classifications of HNSCC have described different head and neck tumor subgroups [5,6,7,8,9] (for a recent review, see [17]), that have been given different names. We showed that there are common subgroups with similar molecular identities, when considering four different datasets [5,6,7,8] (Supplementary Materials Figure S1A). We focused on one subgroup, named “Basal” in [6,7,8] and “Group1” in [5], which comprises about 30% of HNSCC tumors. These tumors are mainly located in the oral cavity and to some extent in the oropharynx, HPV-negative, and composed of well-differentiated tumors (Figure 1A). Signaling pathway analysis across the public datasets established that basal tumors display up-regulation of genes involved in the EGFR signaling pathway (*amphiregulin* (*AREG)*, *EREG*), adaptation to hypoxia (*Hypoxia Inducible Factor- 1α* (*HIF1A)*, *Carbonic Anhydrase 9 (CA9*)), and differentiation/keratinization of basal epithelial cells (*Cadherin 3* (*CDH3)*, *Cadherin 13* (*CDH13*)).

We investigated whether the same subgroups could be identified in our transcriptomic analysis of 98 HNSCC samples [18]. We found four equivalent expression subgroups in our collection (i.e., atypical (*n* = 28/98), basal (*n* = 40/98), classical (*n* = 17/98), and mesenchymal (*n* = 11/98) tumors), and confirmed the presence of the basal subgroup by analyzing characteristic overexpressed genes. We initially identified 18 genes that were up-regulated in the basal subgroups (Supplementary Materials Table S1) of the three available datasets [6,7,8]. They included *EREG* and *AREG*, which encode ligands of the EGF receptor (epiregulin and amphiregulin, respectively) [14,19]. Using RT-qPCR-based gene expression assays on RNA extracted from the 98 tumor samples, we found a statistically significant higher expression of both *AREG* and *EREG* in the basal tumors (ANOVA *p* < 0.001; Figure 1B). Receiver Operating Characteristic (ROC) curve analysis was used for these two genes to determine the area under the curve (AUC; Figure 1C). The AUCs for *AREG* and *EREG* were 0.911 and 0.858, respectively, indicating their strong relationship with the tumor subgroup. These observations confirm that the expression of factors involved in EGF receptor signaling is a characteristic feature of the basal molecular subgroup of HNSCC.

### 2.2. Basal-Like Cell Lines Are More Sensitive to Pharmacological EGFR Blockade

**Figure 2:**
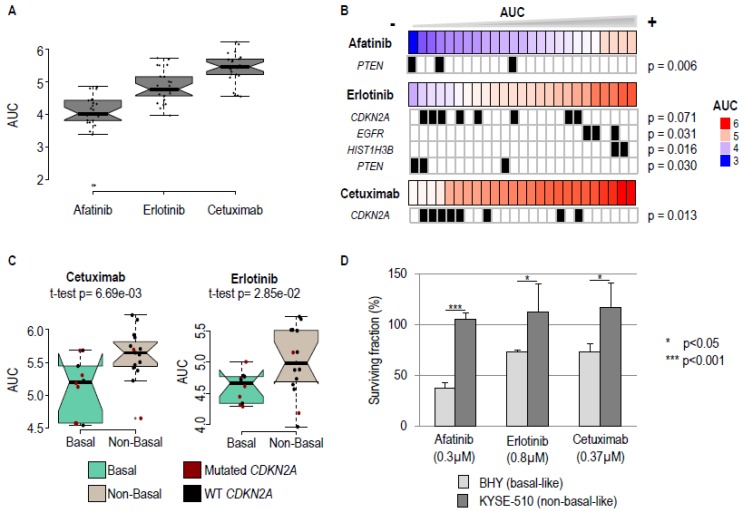
Basal-like HNSCC cells are sensitive to epidermal growth factor receptor (EGFR) blockade. (**A**) Boxplot of the AUC for the 25 HNSCC cell lines treated with the three anti-EGFR drugs (Afatinib, Erlotinib, and Cetuximab). (**B**) Representation of the clinically relevant mutations association with response to Afatinib, Erlotinib, and Cetuximab. For each drug, cell lines are ordered by increasing AUC. *p*-values correspond to moderate *t*-test *p* values, comparing AUC between mutated and wild type (WT) cell lines. (**C**) Boxplot representation of the AUC obtained after Cetuximab and Erlotinib treatment of basal and non-basal HNSCC cell lines. The AUC were found to be significantly lower (*t*-test *p*-values are shown) in basal-like cells. (**D**) Analysis of the clonogenic survival of basal BHY and non-basal KYSE-510 cells upon treatment with Afatinib, Erlotinib, and Cetuximab. BHY cells display a significantly higher sensitivity to EGFR blockade. Mean survival fractions and standard errors from three independent experiments are shown.

Several studies indicate that patients with basal tumors could be more sensitive to EGFR-targeted treatments [9,10]. We found, from the analysis of public data bases, that basal-like HNSCC cell lines appear to be more sensitive to EGFR-targeted therapy. An examination of data from the Genomics Drug Sensitivity in Cancer project showed that the half maximal inhibitory concentration (IC_50_) values for Gefitinib (a tyrosine kinase inhibitor, TKI) and for Cetuximab (an anti-EGFR antibody) are significantly lower in basal as compared to non-basal cells (Supplementary Materials Figure S2A,B). In addition, analysis of the Cancer Cell Line Encyclopedia (CCLE) showed that the IC_50_ for the TKIs Erlotinib and Lapatinib are lower in the basal subgroup (Supplementary Materials Figure S2C,D). However, these studies are not directly comparable, since different drugs and samples (cell lines versus tumor specimens) were tested. We addressed this issue using a high-throughput comprehensive approach. We initially classified cell lines into basal and non-basal subtypes (Supplementary Materials Figure S3) using a pattern of gene expression that categorized tumor samples into the corresponding subtypes and gene expression data from the Genomics Drug Sensitivity in Cancer project [20] and the CCLE [21] databases. We selected 10 basal-like and 15 non-basal-like HNSCC cell lines (Supplementary Materials Table S2). These cell lines were screened for their in vitro sensitivity to three drugs that target EGFR (i.e., Cetuximab, Erlotinib, Afatinib; see Material and Methods). Standard dose-response curves were established for all of the drugs and cell lines, and the areas under the curve (AUC), which inversely correlate with drug sensitivity, were used to evaluate drug response (Table 1). Afatinib was found to be the most efficient and Cetuximab the least efficient, for all of the cell lines (Figure 2A; Table 1). The effects of the two tyrosine kinase inhibitors, Erlotinib and Afatinib, correlated more closely to each other than to Cetuximab, as expected from their different modes of action (Supplementary Materials Figure S4).

In order to evaluate the impact of oncogenic drivers on EGFR blockade, the cell lines were screened for mutations in 26 genes that are routinely investigated in patient tumors (Supplementary Materials Table S3), and the association between mutational status (Supplementary Materials Table S4) and drug resistance/response was investigated. Mutations in four genes (*CDKN2A*, *Phosphatase And Tensin Homolog* (*PTEN*), *EGFR,* and *Histone Cluster 1 H3 Family Member B* (*HIST1H3B*) were found to be associated with response (Figure 2B; *p* values < 0.1). Mutations in three genes, *PTEN*, *EGFR,* and *CDKN2A*, are associated with an improved drug response (smaller AUC), whilst a mutation in the histone *HIST1H3B* gene favors drug resistance. Mutations in *CDKN2A* are the most frequent (8/25 cell lines), and are mainly found in basal cell lines (6/10 basal cell lines, chi² *p* value = 0.044). Nevertheless, basal status is a better discriminator of response to Erlotinib or Cetuximab than *CDKN2A* mutations (Figure 2C; compare basal-like and non-basal-like tumors in green and grey).

In order to corroborate the results of the screen, we selected the BHY and KYSE-510 cell lines to represent the basal and non-basal subgroups, respectively, based on their characteristic differential sensitivity to EGFR blockade (Supplementary Materials Figure S5). We used drug concentrations that are likely to have different effects on the two subgroups (Supplementary Materials Figure S5), and *AREG* and *EREG* RNA expression and cell survival as readouts. As expected, *AREG* and *EREG* RNAs were found to be more highly expressed in BHY compared to KYSE-510 (Supplementary Materials Figure S6A, see the zero time point and above). Activation of the EGFR pathway with EGF consistently upregulated expression of both *RNAs* (Supplementary Materials Figure S6A), showing that this pathway can be modulated in both cell lines. Treatment with 0.3 µM Afatinib, 0.8 µM Erlotinib, or 0.37 µM Cetuximab for 24, 48, 72, and 96 h significantly reduced the expression of *AREG* and *EREG* in BHY, but not in KYSE-510 (Supplementary Materials Figure S6B). The same treatments significantly reduced clonogenic survival of BHY, but not of KYSE-510 (Figure 2D). Altogether, these results confirm that the pharmacological blockade of EGFR preferentially inhibits the EGFR signaling pathway and cell survival of basal HNSCC cell lines.

### 2.3. Greater Response of Basal Cell Lines to Certain Drug Combinations

Since cancer therapies often require drug combinations to increase efficacy (e.g., [3,12]), we investigated whether the EGFR blockade could be enhanced with clinically relevant inhibitors of associated pathways, including the dual PI3K/mTOR inhibitor Gedatolisib, the MEK inhibitor Cobimetinib, and the anti-HER2/Erbb2 receptor tyrosine kinase Trastuzumab monoclonal antibody [22,23]. Gedatolisib was found to be the most efficient, with a mean AUC of ~3, whereas Cobimetinib or Trastuzumab gave mean AUCs >4 (Supplementary Materials Figure S7A). The correlations between the AUCs were found to be relatively low, consistently targeting different pathways (Supplementary Materials Figure S7B). Mutations in the *CDKN2A*, *PIK3CA*, *PTEN,* and *NRAS* genes were, to some extent, associated with increased drug sensitivity (Supplementary Materials Figure S7C, *p* < 0.1)). However, basal status was more significantly correlated with response to Cobimetinib than *CDKN2A* and *NRAS* mutations (Supplementary Materials Figure S7D; compare basal-like and non-basal-like tumors in green and grey).

**Figure 3:**
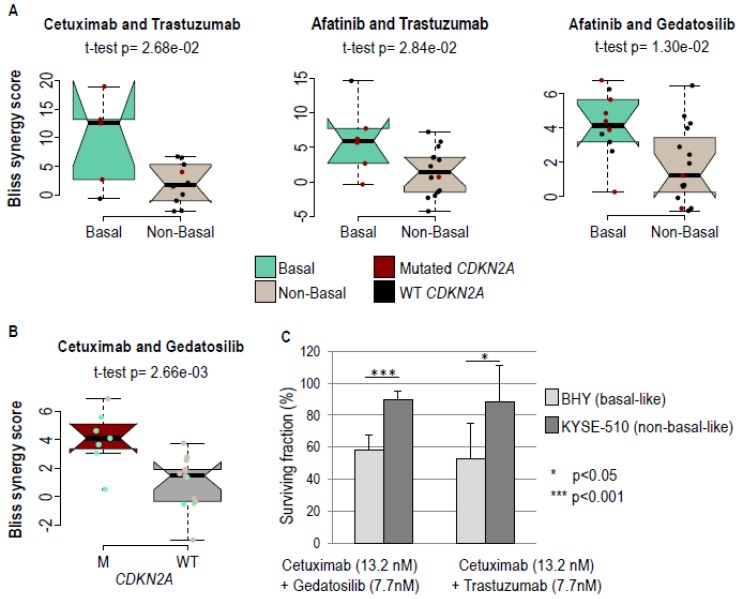
Basal HNSCC cells are sensitive to therapeutic combinations. (**A**) Boxplot of the Bliss synergy scores obtained on basal and non-basal HNSCC cell lines upon treatment with the shown therapeutic combination (Cetuximab and Trastuzumab; Afatinib and Trastuzumab; Afatinib and Gedatosilib). The Bliss scores were found to be significantly higher (*t*-test *p*-values are shown) in basal cells. (**B**) Boxplot of the Bliss synergy scores obtained on wild-type (WT) and *CDKN2A* mutant (M) HNSCC cell lines upon treatment with Cetuximab and Gedatosilib. *CDKN2A* mutations are significantly associated with higher Bliss scores. (**C**) Analysis of the clonogenic survival of basal BHY and non-basal KYSE-510 cells upon treatment with Cetuximab and Gedatosilib, and with Cetuximab and Trastuzumab. BHY cells displayed a significantly higher sensitivity to therapeutic combinations. Mean survival fractions and standard errors from three independent experiments are shown.

Gedatolisib, Trastuzumab, and Cobimetinib were then tested in combination with the three anti-EGFR drugs (i.e., Cetuximab, Erlotinib, Afatinib). Bliss synergy scoring models were used to compare the efficiencies of these nine combinations (Table 2; note that 0 Bliss synergy scores indicate no synergy, high scores synergy, and negative scores antagonism). Six combinations were found to be synergistic with all of the cell lines (Supplementary Materials Figure S8; red brackets), namely the three combinations with Trastuzumab and with Gedatosilib. Basal status was associated with synergy for three combinations, Cetuximab with Trastuzumab, Afatinib with Trastuzumab, and Afatinib with Gedatolisib (Figure 3A; compare basal-like and non-basal-like tumors in green and grey). Mutation of the *CDKN2A* gene was statistically associated with the synergy of Cetuximab and Gedatosilib (Figure 3B; compare wild type and mutated *CDKN2A* tumors in black and red). We validated these results with clonogenic survival assays on selected representative cell lines (BHY and KYSE-510; see above) and discriminatory drug combinations (13.2 nM Cetuximab + 7.7 nM Gedatosilib, or 13.2 nM Cetuximab + 7.7 nM Trastuzumab) identified with the high throughput assay. BHY basal-like cells had a significantly higher sensitivity to these therapeutic combinations than the KYSE-510 non-basal-like cells (Figure 3C).

### 2.4. EREG Downregulation in the Basal-Like BHY Cell Line Inhibits Cell Survival

**Figure 4:**
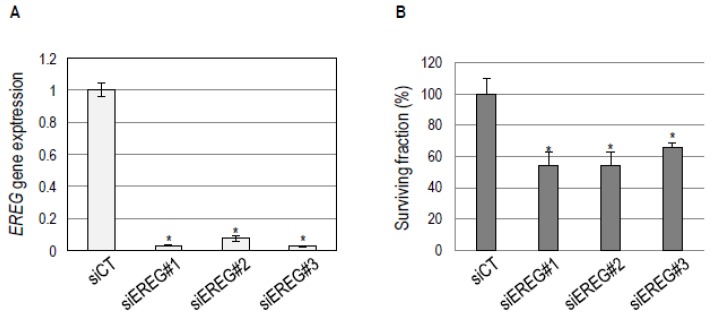
*EREG* expression inhibition decreases BHY basal cell clonogenic survival. (**A**) RT-qPCR analysis of the expression of the *EREG* gene in BHY cells transfected with three independent anti-*EREG* siRNAs (si*EREG*#1, 2, and 3). Cells transfected with scrambled siRNA were used as a negative control (siCT). Transfection with si*EREG* siRNAs yielded a significant >90% inhibition of *EREG* expression. (**B**) Analysis of the clonogenic survival of BHY basal cells upon *EREG* expression inhibition. Transfection with si*EREG*#1, 2, and 3 significantly reduces BHY cell clonogenic survival. Cells transfected with scrambled siRNA were used as a negative control (siCT). Mean survival fractions and standard errors from three independent experiments are shown. *: *p* < 0.05.

Our observations that the EGFR blockade inhibits both *EREG* expression and cell survival in a subgroup-specific manner (Supplementary Materials Figure S6; Figure 2; Table 1) suggest that *EREG* expression could, in itself, be important for the survival of basal HNSCC cells. To test this hypothesis, we studied the effects of *EREG* knockdown on the survival of BHY basal cells. Three independent anti-*EREG* siRNAs were selected that inhibit *EREG* expression by >90% (Figure 4A). Interestingly, *EREG* inhibition decreased the clonogenic survival of BHY cells by almost 50% (Figure 4B). These observations suggest that basal HNSCC could be addicted to an *EREG* feedback loop and that *EREG* expression could be a “functional” biomarker for HNSCC sensitivity to the EGFR blockade (see Discussion).

## 3. Discussion

HNSCC tumors can be divided into four groups based on their molecular characteristics: classical, basal, mesenchymal, and atypical [5,6,7,8,9,18]. Interestingly, basal tumors overexpress genes linked to activation of the EGFR pathway, indicating sensitivity to inhibition of EGFR [9,10]. We confirmed this hypothesis with a collection of HNSCC cell lines that resemble basal or non-basal tumors, based on their molecular features reported in several databases (the Genomics of Drug Sensitivity in Cancer (GDSC) project [11] and/or the Cancer Cell Line Encyclopedia [21]). Cell lines were treated with the small TKIs Erlotinib and Afatinib, as well as the anti-EGFR monoclonal antibody Cetuximab. Erlotinib and Afatinib have lower AUCs and more closely related efficacies compared to Cetuximab. This might be expected from their molecular nature and mode of action (TKI versus monoclonal antibody). Afatinib is more efficient than Erlotinib (both lower AUC and survival fraction), as would be expected from its modes of inhibition (irreversible adduct versus competitive inhibitor, respectively). The higher efficacy of TKIs in vitro apparently conflicts with clinical observations, where anti-EGFR TKIs have yielded disappointing results. Erlotinib monotherapy of patients with recurrent/metastatic HNSCC showed a 4% response rate [24], and Afatinib did not improve overall survival in the phase III LUX-Head and Neck 1 trial [25]. Erlotinib combined with chemoradiotherapy compared to chemoradiotherapy alone did not improve tumor response and progression-free survival in patients with recurrent/metastatic (R/M) HNSCC [26]. These discrepancies can be interpreted in several ways. The clinical efficacy of Cetuximab partly relies on antibody-dependent cell cytotoxicity by natural killer (NK) cells [27,28], which is not represented in our in vitro assays. In addition, patients were not stratified into molecular subtypes, which might introduce a bias, since the potentially sensitive subgroup is relative small (~30% are of the basal type). Indeed, patients stratified by EGFR overexpression had improved objective response rates, disease control rates, and median progression-free survival after Afatinib treatment compared to methotrexate, in the LUX-Head and Neck 1 trial [29]. These findings suggest that tumor molecular features could be used to guide treatment strategies for HNSCC patients.

In order to increase the efficacy of the EGFR blockade, we tested combinations with inhibitors of downstream pathways. The PI3K/mTor inhibitor Gedatolisib was more efficient than any of the other treatments. Interestingly, Gedatolisib, in combination with the CDK4/6 Inhibitor Palbociclib, is being evaluated for patients with various solid tumors (including head and neck cancer), in the NCT03065062 phase I trial (ClinicalTrial.gov reference) that is currently recruiting participants. We tested a combination with a HER-2 inhibitor (Trastuzumab) because there is some evidence that HER-2 overexpression is associated with poor outcomes in HNSCCC [30,31] and that HER-2 can affect the response to the EGFR blockade. HER-2 can affect signaling by forming heterodimers with other members of the ErbB family [32]. EGFR/HER2 heterodimers decrease the efficacy of EGFR blockade in HNSCC cell lines [33,34]. In addition, acquired Cetuximab resistance in HNSCC xenografts can be overcome with a dual EGFR-HER2 kinase inhibitor [35].

In our screen, the Afatinib/Trastuzumab combination was found to be efficient on basal-like HNSCC cell lines. Interestingly, inhibition of both EGFR and HER2 with a Cetuximab/Trastuzumab combination has been shown to preferentially inhibit 8 out of 16 HNSCC cell lines [36]. In addition, adjuvant dual HER-2 blockade using Trastuzumab and Lapatinib (in combination with anthracycline/taxane–based chemotherapy) significantly increased the rate of the pathological complete response (pCR) of HER-2 positive primary breast tumors to over 50% compared to Trastuzumab provided alone [37]. When Afatinib was tested in a similar setting, pCR was achieved in 49% of the cases [38]. Drug combinations can have additional effects in tumors that are not reflected in our in vitro system. For example, TKIs are known to increase Trastuzumab-dependent cell mediated cytotoxicity [39,40].

**Figure 5:**
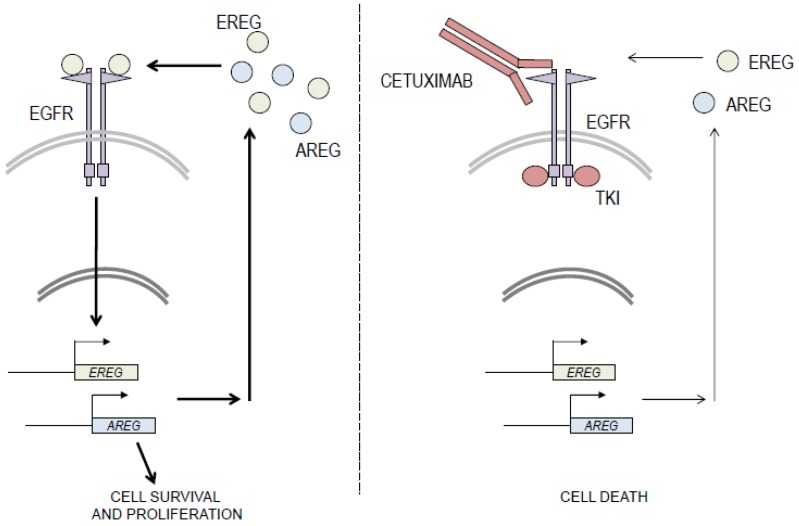
Model for the oncogenic activation of the EGFR signaling pathway and response to therapeutic EGFR-blockade in basal HNSCC. Our results suggest that basal HNSCC cell proliferation and survival are activated by an oncogenic positive feedback loop that is fueled by the expression of EGFR ligands, including epiregulin, and the activation of the EGFR signaling pathway. Pharmacologic blockade of EGFR with monoclonal antibodies or TKIs results in down-regulation of ligand gene expression and diminution of the activity of the EGFR signaling pathway, leading to a decrease in cell viability.

We propose that the sensitivity of basal-like cells to EGFR blockade results from their addiction to an oncogenic auto-amplifying loop that is characterized by high-level expression of epiregulin and amphiregulin (see Figure 5). This hypothesis is supported by both our data and the literature.

We found that EGFR blockade efficiently downregulated transcripts for the EGFR ligand epiregulin in basal-like HNSCC cells, and reduced their clonogenic survival. Direct downregulation of *EREG* transcripts with three different siRNAs reduced the clonogenic survival of BHY basal cells. We also found that EGFR blockade (see Results) as well as downregulation of *EREG* transcripts with siRNAs [41] inhibited *AREG* expression, suggesting that *EREG* and *AREG* expression may be cross-regulated. Our in vitro results agree with other studies. Oliveras-Ferraros et al. [42] showed that sensitivity to Cetuximab depends on high expression of *EREG* and *AREG*, and that *AREG* and *EREG* cross-regulate. Oshima et al. [43] found that the response of HNSCC cell lines to EGFR blockade correlates with the expression levels of several EGFR ligands. Jedinski et al. [44] reported a connection between *EREG* mRNA expression and response to Cetuximab using a collection of HNSCC cell lines. Using clinical data and a meta-analysis, high *AREG* and *EREG* mRNA expression levels were found to be associated with both progression-free and overall survival of patients with metastatic colorectal cancer treated with Cetuximab-based chemotherapy [45]. Similarly, high tumor *EREG* mRNA expression was found to be associated with improved prognosis upon Cetuximab treatment, in a randomized phase III clinical trial [46]. Furthermore, high *EREG* and *AREG* expression was recently reported to correlate with longer progression-free and overall survival in a retrospective series of patients with recurrent/metastatic HNSCC treated with Cetuximab and chemotherapy [47]. Altogether, these observations suggest that high EREG/AREG expression is a predictive functional marker of oncogenic addiction that warrants further evaluation, especially for its value in the clinic.

## 4. Materials and Methods

### 4.1. Cell Lines

25 HNSCC cell lines that have been characterized by the Genomics of Drug Sensitivity in Cancer project of the Sanger Centre [20] were procured from the corresponding sources, including cell-line collections and individual scientists, or were already available from the Institut de Génétique et de Biologie Moléculaire et Cellulaire (IGBMC) cell-culture facility. Their identities were verified, either from the certificate supplied by the vendor/distributor, or using the American Type Culture Collection (ATCC) service (authentication by short tandem repeat profiling). Two cell lines did not match standard cell-lines available from public collections, as expected from their private source. All the cell-lines were verified for absence of mycoplasma. Cell lines without a vendor certificate of mycoplasma status were tested for contamination by the IGBMC service. If necessary, cell lines were decontaminated and re-verified for authenticity. For screening purposes, the cell culture conditions were reduced to a minimum set used by the Sanger Centre. Most of the cell lines were found to grow readily in one of two media. Frozen stocks of the cell lines in “screening media” were made.

### 4.2. Reagents

Chemicals (Cobimetinib, Afatinib, Erlotinib, Gedatosilib) were purchased from SelleckChem and antibodies (Cetuximab and Trastuzumab) were obtained from the Pharmacy Department of the Paul Strauss Cancer Center (CLCC, Nelly Etienne-Selloum). BHY and KYSE-510 cells at 70% confluency were grown with 10, 100, and 1000 ng/mL human epidermal growth factor (E9644-.2MG Sigma, Saint-Quentin-Fallavier, France) for 24 h.

### 4.3. Small Interfering RNA Transient Transfection

Three independent small interfering RNAs targeting *EREG* (siGENOME Human EREG (2069) siRNA-Individual, D-011268-01-0005 and D-011268-02-0005, Dharmacon; EREG MISSION Pre-designed siRNA, SASI_Hs02_00331836, Sigma) were transfected to BHY cells using Lipofectamine RNAiMAX, according to the manufacturer’s instructions. Total protein and RNA was extracted 72 h post-transfection to assess EREG inhibition (see below). Transfected cells were seeded 72 h post-transfection for clonogenic survival assays (see below).

### 4.4. Single-Drug and Drug-Combination Dose-Response Analyses

For each cell line, seeding conditions were determined in 384-well microplates in order to reach 90% confluency at day four, whilst maintaining exponential growth. For dose-response analyses, cells were seeded (day 0) and incubated for 24 h before adding 13 concentrations of each of the six compounds (0.5 nM-30 µM, 2.5-fold dilution for the drugs; 0.1 nM-5.7 µM, 2.5-fold dilutions for Cetuximab; 0.4 nM-24 µM, 2.5-fold dilutions for Trastuzumab). At day four, cells were incubated with the Prestoblue cell viability reagent (Thermofisher Scientific, Illkirch-Graffenstaden, France) for one hour. Technical triplicates were performed. Quantitation of fluorescent signal intensities was performed using the Berthold MITHRAS LB 940 reader at excitation and emission wavelengths of 535/595 nm. Curve fitting of the dose-response data was performed to determine several descriptive parameters, including IC50, area under the curve (AUC), and the fitting correlation parameter of the dose response curve. The AUC was used to analyze the results.

For analysis of drug combinations, dose-response matrix data (single replicate) were obtained using six concentrations of each compound (1.3 nM-10 µM, 6-fold dilutions for the drugs; 0.37 nM-2.85 µM, 12-fold dilutions for Cetuximab; 1.54 nM-12 µM; 12-fold dilutions for Trastuzumab). As described above, cells were incubated at day four with the cell viability reagent and fluorescent signal intensities acquired. Curve fitting and synergy scoring (Bliss reference model) were done with SynergyFinder [48].

### 4.5. Next Generation Sequencing

DNA was extracted from cultured cells (5 million cells harvested in 200 µL PBS) using the QIAamp DNA Mini Kit (Qiagen, Courtaboeuf, France). Double strand DNA was quantified using a fluorometric method (Qubit dsDNA BR Assay, Thermo Fisher Scientific) and qualified by real-time PCR (FFPE QC Kit, Illumina, Paris, France). Mutation screening was performed by next generation sequencing (NGS) on a MiSeq Illumina platform using the Tumor Hotspot MASTR Plus assay (Multiplicom-Agilent, Les Ulis, France), which targets frequently occurring mutations (hotspots) in a panel of 26 genes (Supplementary Materials Table S1). Sequencing data were aligned to human genome hg19 using the BWA-MEM algorithm (Burrows–Wheeler Aligner-maximal exact matches). Variants were called using three different variant callers: VarScan, GATK HaplotypeCaller (GATK-HC), and GATK Unified Genotyper (GATK-UG). The minimum coverage per base (DP for depth) and the minimum variant allelic frequency (VAF) were fixed at 300-fold (300×) and 4% respectively, which allowed a sensitivity of 100% in the detection of mutations in the internal positive control (Tru-Q3 DNA from Horizon Diagnostics) included in each sequencing run. Reported mutations corresponded to splice site or non-synonymous exonic variants, excluding known polymorphisms, located in the genes and exons of Supplementary Materials Table S1.

### 4.6. Datasets

Four public datasets of transcriptomic profiles of HNSCC tumors were used: the 60 transcriptomic profiles of Chung et al. [5] (GSE686), the 138 transcriptomic profiles of Walter et al. [6] (GSE39368), the 303 transcriptomic profiles of the Cancer Genome Atlas [7], and the 134 transcriptomic profiles of Keck et al. [8] (GSE40774). Two public datasets of transcriptomic profiles of HNSCC cell lines were studied: the 51 HNSCC cell lines of the Cancer Cell Line Encyclopedia (CCLE) [21] and the 47 HNSCC cell lines of the Genomics Drug Sensitivity in Cancer (GDSC) project [20]. The transcriptomic profiles of our 98 HNSCC tumor samples [18] were used to predict the basal subtype in our tumor collection (E-MTAB-1328).

### 4.7. Data Analyses

We used ANOVA models and the moderate *t*-test to identify differentially expressed genes. The association between the partitioning of the samples and the bio-clinical factors was evaluated in chi-squared tests or Fisher-exact tests when required. We used hypergeometric tests to measure the association between a gene list and the 17,306 biological pathways collected from Kyoto Encyclopedia of Genes and Genomes (KEGG), MSigDB, Gene Ontology (GO), or Biocarta. The four public datasets of HNSCC tumors originated four HNSCC molecular classifications [5,6,7,8]. Centroids were built for each of these subtypes with the common differentially expressed genes. Pearson correlations were used to compare the resulting centroids. The four transcriptomic datasets were scaled and aggregated. A centroid-based predictor was then built on this aggregated dataset to predict the basal subtype in the two transcriptomic datasets of cell lines [20,21]. A cell line was defined as basal if the prediction was basal in the two datasets, and a cell line was defined as non-basal if the prediction was non-basal in the two datasets, or if the prediction was non-basal in one dataset and the cell line was not present in the second dataset. The criteria of fold change and area under the curve (AUC) were used to select the best markers of the basal subtype.

### 4.8. Clonogenic Survival Assays

Cultured BHY and KYSE-510 cells in exponential growth were trypsinated and seeded in 6-well plates at two different concentrations (BHY: 250 and 400 cells/well; KYSE-510: 100 and 200 cells/well). Twenty-four hours after dilution, cells were grown in the presence of drugs (0.3 µM Afatinib; 0.8 µM Erlotinib; 0.37 µM Cetuximab; 13.2 nM Cetuximab + 7.7 nM Gedatosilib; 13.2 nM Cetuximab + 7.7 nM Trastuzumab) for 96 h. After replacement with fresh culture medium, cells were grown for 12 days to allow colony formation. Clones were stained with 0.05% crystal violet (Sigma Aldrich, Lyon, France) in 5% ethanol, and positive colonies (>64 cells) were counted. The plating efficiency was calculated by dividing the number of positive colonies that grew in the absence of treatment, divided by the number of cells that were seeded. The surviving fraction was calculated by dividing the number of positive colonies upon treatment by the number of cells that were seeded, multiplied by the plating efficiency.

### 4.9. Gene Expression Assays

Gene expression assays in tumor samples were performed by extracting the total RNA from 98 HNSCC frozen tissues [18] using the RNeasy kit (Qiagen, Courtaboeuf, France), according to the manufacturer’s instructions. The integrity of extracted RNA was verified on an Agilent 2100 Bioanalyser (Agilent Technologies, Palo Alto, CA, USA). RNA was retro-transcribed using the Goscript reverse transcription system (Promega, Charbonnières-les-Bains, France), and real-time quantitative PCR was performed using the LightCycler^®^ 480 real-time PCR system (Roche, Mannheim, Germany). qRT-PCR data were analyzed using the LightCycler^®^ 480 software. The expression levels of each gene were normalized to the geometric mean Ct values of two internal controls, Ribosomal Protein Long P0 (RPL0) and Ubiquitin B (UBB). The following primer pairs were used: AREG (5′-CCACAGTGCTGATGGATTTG-3′ and 5′-GCCAGGTATTTGTGGTTCGT-3′), EREG (5′-TCCCAGGAGAGTCCAGTGAT-3′ and 5′-GTGTTCACATCGGACACCAG-3′), RPLP0 (5′-GAAGGCTGTGGTGCTGATGG-3′ and 5′-CCGGATATGAGGCAGCAGTT-3′) and UBB (5′-GCTTTGTTGGGTGAGCTTGT-3′ and 5′-CGAAGATCTGCATTTTGACCT-3′).

Gene expression assays on cultured cells were performed by extracting total RNA from pelleted cells using a standard TRIZol procedure (TRI Reagent^®^: TR 118 Molecular Research Center), according to manufacturer’s instructions. RNA was retro-transcribed using the Goscript reverse transcription system (Promega), and real-time quantitative PCR was performed using the LightCycler^®^ 480 real-time PCR system (Roche). *AREG* and *EREG* expression was measured using the pairs of primers shown above, and normalized to the expression of 18S RNA (ARN18S Forward: 5′-tgtggtgttgaggaaagcag-3′; ARN18S Reverse: 5′-tccagaccattggctaggac-3′) using the 2^−ΔΔCt^ method.

## 5. Conclusions

This manuscript contributes a new comprehension of the molecular heterogeneity of head and neck squamous cell carcinoma (HNSCC) and finds a way of potentially exploiting this heterogeneity through the demonstration that one subgroup (basal) is particularly sensitive to the EGFR blockade. It proposes a molecular mechanism to explain this “oncogenic addiction” that could be the basis of not only further studies of a novel mechanism, but also improvements in the clinical management of this dreadful disease.

## Figures and Tables

**Table 1:** Analysis of the response of basal-like and non-basal like HNSCC cell lines to treatment with Afatinib, Erlotinib, Cetuximab, Gedatolisib, Cobimetinib, and Trastuzumab. Cell line names and subgroup (basal versus non-basal), drug names, and AUC are shown.

Cell Line	Molecular Subtype	AUC					
		Afatinib	Erlotinib	Cetuximab	Gedatolisib	Cobimetinib	Trastuzumab
BHY	Basal	3.467751696	4.286063175	4.57727436	3.302462568	4.608746135	4.69416933
CAL27	Basal	3.814815676	4.444098626	5.680773203	3.184901695	3.747852593	5.5725818
CAL33	Basal	3.744753557	4.310451267	4.573913673	2.406624054	4.450866901	5.076910559
HSC2	Basal	4.3439915	4.741863856	5.127815501	2.566283234	4.420333039	4.946942339
HSC3	Basal	3.957972571	4.6095489	5.183631235	2.952916999	3.8894347	5.295803542
KYSE150	Basal	4.441065136	4.780103002	5.684081754	2.980021031	4.687959978	5.550264191
SCC15	Basal	4.315046458	4.766164343	4.54432253	3.764267942	4.6050741	5.494692438
SCC25	Basal	4.177447191	4.336103828	5.446338918	2.771755456	3.798183465	5.231781001
SCC4	Basal	4.019642792	5.002304825	5.305982451	2.737492906	4.32226842	5.256117469
SCC9	Basal	4.134972926	4.713456974	5.222760141	3.005065893	4.873465213	4.87701988
KYSE270	Non-Basal	3.805157987	5.500896683	5.647765664	3.332916543	5.232659439	5.565586558
KYSE180	Non-Basal	4.01491891	4.729217951	5.705364107	3.147861975	4.918800553	5.272014082
TE6	Non-Basal	4.258968922	4.879974238	5.750123119	3.639308374	5.517244329	4.979449358
TE5	Non-Basal	3.9331781	5.159062006	5.461107055	2.778634257	4.731403338	5.23314044
TE10	Non-Basal	4.006096215	5.513704267	5.222443794	3.497187601	4.890993502	4.946552101
KYSE140	Non-Basal	3.375761348	4.562986869	5.376787765	2.919235293	4.740974506	5.268327679
KYSE510	Non-Basal	4.845901636	5.515376689	5.879640984	3.013249683	5.011478461	5.476614796
RPMI2650	Non-Basal	4.780339567	5.726684777	6.149644208	3.600359879	5.476229419	5.540633467
LB771HNC	Non-Basal	4.793902186	5.152157915	5.694559888	3.158211825	5.585646234	5.618555173
HCE4	Non-Basal	4.424539135	5.687089202	5.91969998	3.64854173	4.638847027	5.069966605
KYSE70	Non-Basal	4.46079496	4.986291447	5.50519843	3.657912306	4.8211094	5.244702124
TE4	Non-Basal	1.814915762	3.96069768	5.420791576	2.170670697	4.2463357	5.08505704
TE14	Non-Basal	3.746428006	4.638733677	5.619299993	3.187628054	4.881707599	4.869384943
TT	Non-Basal	4.822344476	4.872688138	6.220243167	3.678272613	4.728732451	5.642166616
HSC4	Non-Basal	3.655491187	4.180726139	4.651136469	2.751213387	3.871130995	5.136060907

**Table 2:** Evaluation of the therapeutic efficiency of drug combinations, as determined by Bliss synergy scoring models. The Bliss score for each drug combination is shown for the 25 HNSCC cell lines.

Cell Line	Molecular Subtype	Bliss Synergy Score								
		Afatinib Cobimetinib	Afatinib Gedatosilib	Afatinib Trastuzumab	Cetuximab Cobimetinib	Cetuximab Gedatosilib	Cetuximab Trastuzumab	Erlotinib Cobimetinib	Erlotinib Gedatosilib	Erlotinib Trastuzumab
BHY	Basal	3.777	5.658	−0.348	7.32	5.569	13.195	4.33	4.807	1.701
CAL27	Basal	6.881	6.787	6.243	4.252	3.649	18.943	−0.354	0.38	−0.958
CAL33	Basal	1.89	0.267	5.684	0.134	4.621	12.537	−4.981	2.219	−2.872
HSC2	Basal	−4.527	4.403	NA	NA	3.066	NA	−0.745	2.266	4.648
HSC3	Basal	−3.278	3.889	7.754	0.688	4.096	NA	0.517	1.721	−0.263
KYSE150	Basal	−0.552	2.649	NA	5.502	−0.469	−0.595	−1.648	−0.445	1.263
SCC4	Basal	5.145	4.867	2.687	1.146	0.522	2.728	2.702	2.979	−1.275
SCC9	Basal	−3.499	6.269	NA	−1.755	1.367	NA	−1.792	2.185	NA
SCC15	Basal	−3.148	3.646	14.661	5.549	−0.535	NA	−1.593	0.959	6.95
SCC25	Basal	2.244	3.184	NA	3.195	NA	NA	−0.986	−2.037	NA
HCE4	Non-Basal	−2.168	−0.69	2.283	0.468	NA	−2.688	4.937	−0.317	4.016
HSC4	Non-Basal	−1.389	1.218	3.527	−0.705	6.87	4.059	−5.899	−1.913	−3.647
KYSE70	Non-Basal	0.059	3.988	0.633	−2.264	1.621	1.546	0.589	−1.169	−1.116
KYSE140	Non-Basal	3.049	0.646	7.26	10.795	NA	6.541	0.466	−2.501	5.82
KYSE180	Non-Basal	1.067	2.902	NA	3.957	2.78	NA	1.759	3.795	NA
KYSE270	Non-Basal	−4.853	0.576	−1.243	NA	1.923	−2.788	1.111	−1.681	−3.602
KYSE510	Non-Basal	−0.962	6.481	−1.99	0.86	−0.325	2.142	1.738	4.474	6.968
LB771HNC	Non-Basal	3.722	−0.689	0.706	7.077	NA	NA	−3.277	0.658	−1.902
RPMI2650	Non-Basal	−2.515	4.699	−4.25	NA	NA	NA	NA	NA	NA
TT	Non-Basal	1.248	0.669	3.563	7.21	−0.107	0.137	7.854	1.774	−3.544
TE4	Non-Basal	6.516	4.268	3.194	−4.664	1.476	−0.945	3.363	1.593	7.991
TE5	Non-Basal	1.231	1.934	−2.293	−2.822	3.753	6.738	−1.631	4.127	7.413
TE6	Non-Basal	NA	−0.834	5.833	0.528	2.57	NA	−1.479	−0.38	4.62
TE10	Non-Basal	4.294	−0.084	−1.468	−6.371	−3.032	NA	0.085	0.77	−1.671
TE14	Non-Basal	5.072	2.447	5.155	−5.61	1.86	5.379	−2.549	−1.334	10.887

## Supplementary Materials

The following are available online at https://www.mdpi.com/2072-6694/11/6/795/s1, Figure S1: Heatmap of the correlations of the subtype centroids in four public omic datasets: Chung et al. [5], Walter et al. [6], TCGA [7] and Keck et al. [8], Figure S2: Boxplots of the IC_50_ of Gefinitinib (A), Cetuximab (B), Erlotinib (C) and Lapatinib (D) on basal-like and non-basal-like HNSCC cell lines, determined from the Garnett [20] (A,B) and CCLE (C,D) [21] datasets, Figure S3: Heatmap of the differentially expressed genes between cell lines of the basal and non-basal subtypes in the CCLE [21] and GDSC [20] public datasets, Figure S4: Correlation matrix of the AUC obtained upon treatment with Afatinib, Erlotinib and Cetuximab, Figure S5: Cell viability dose-response curves obtained on BHY (upper panels) and KYSE-510 (lower panels) cells following 96 h of treatment with Cetuximab (left panels), Afatinib (middle panels) and Erlotinib (right panels), Figure S6: Analysis of *AREG* and *EREG* gene expression in BHY and KYSE-510 cell lines, Figure S7: Analysis of the response of the 25 HNSCC cell lines to treatment with Gedatolisib, Trastuzumab and Cobimetinib, Figure S8: Boxplot of the Bliss synergy scores for the 25 cell lines treated with the nine drug combinations, Table S1: List of markers found to be up- and down-regulated in basal HNSCC tumor samples, Table S2: List of the 25 HNSCC cell line, Table S3: List of the 26 clinically relevant cancer mutated genes analyzed in the 25 HNSCC cell lines, Table S4: Results of Next Generation Sequencing of a panel of 26 clinically relevant cancer genes in the 25 HNSCC cell lines.
