# Supplementary material for: Preferential Response of Basal-Like Head and Neck Squamous Cell Carcinoma Cell Lines to EGFR-Targeted Therapy Depending on EREG-Driven Oncogenic Addiction
Source: Cancers (Basel). 2019 Jun 8;11(6):795. doi: 10.3390/cancers11060795 (PMC6627901; doi:10.3390/cancers11060795)
Supplement: Supplementary file 1 [file cancers-11-00795-s001.zip › cancers-508813 - supplementary files/cancers-508813 - Supplementary - final version.pdf]

Supplementary Materials: Preferential Response of Basal-Like Head and Neck Squamous Cell Carcinoma Cell Lines to EGFR-Targeted Therapy Depending on *EREG*-Driven Oncogenic Addiction

Sylvie Job, Aurélien de Reyniès, Betty Heller, Amélie Weiss, Eric Guérin, Christine Macabre, Sonia Ledrappier, Cyril Bour, Christine Wasylyk, Nelly Etienne-Selloum, Laurent Brino, Christian Gaiddon, Bohdan Wasylyk and Alain C. Jung

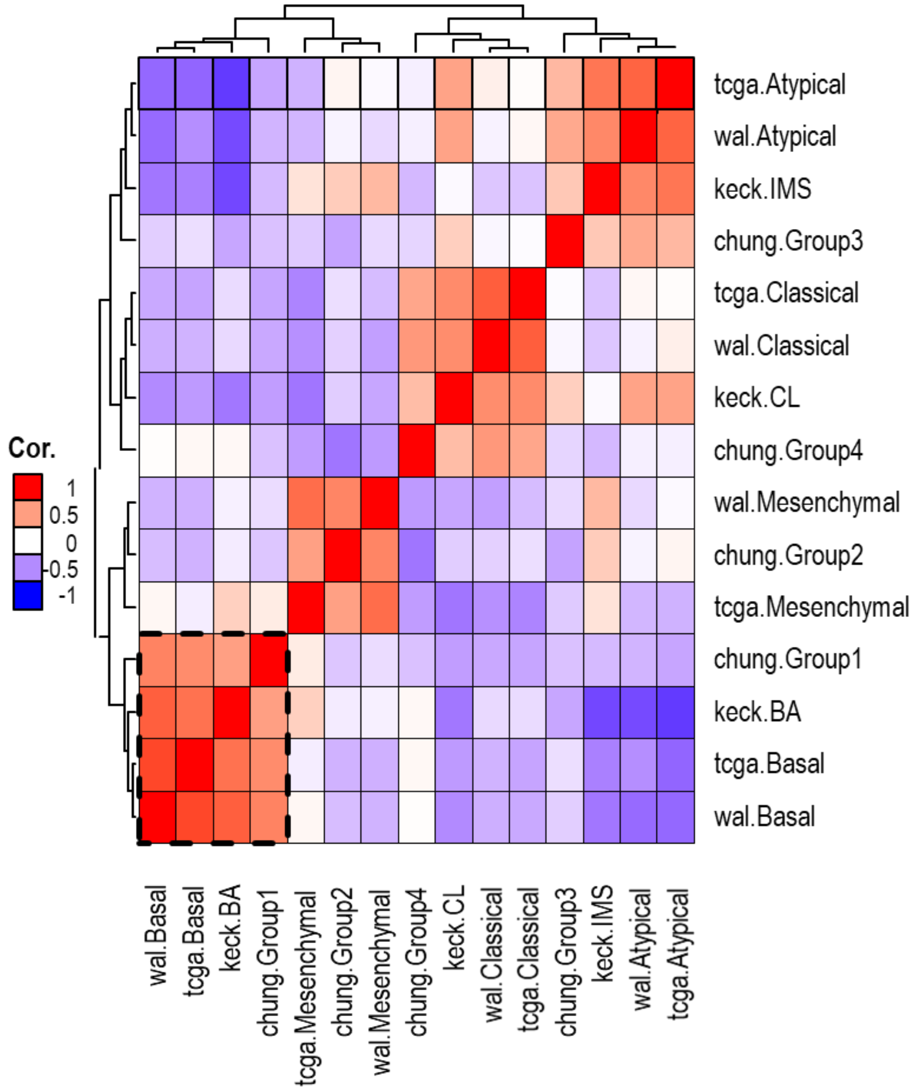

Figure S1. Heatmap of the correlations of the subtype centroids in four public omic datasets: Chung et al. [5], Walter et al. [6], TCGA [7] and Keck et al. [8].

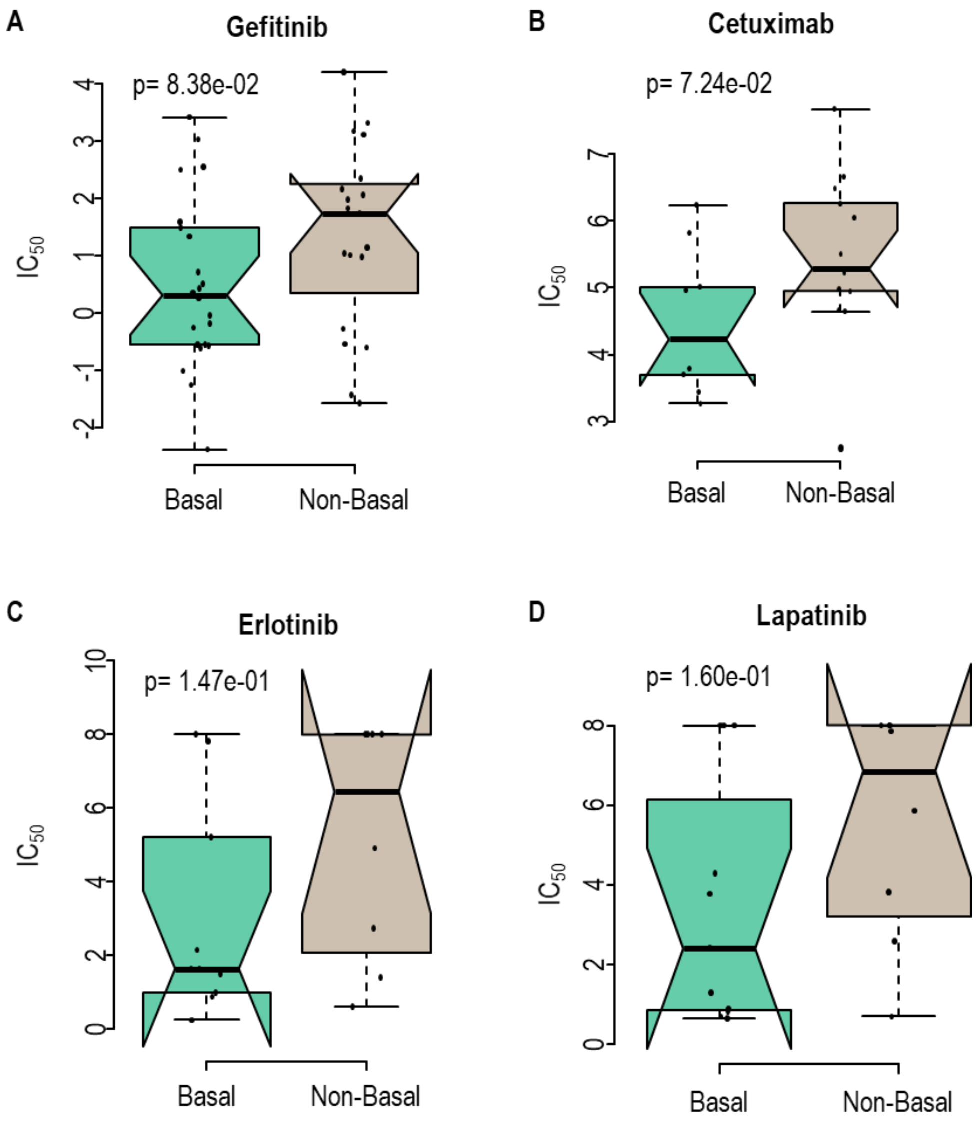

Figure S2. Boxplots of the IC_{50} of Gefinitinib (**A**), Cetuximab (**B**), Erlotinib (**C**) and Lapatinib (**D**) on basal-like and non-basal-like HNSCC cell lines, determined from the Garnett [20] (**A**,**B**) and CCLE (**C**,**D**) [21] datasets.

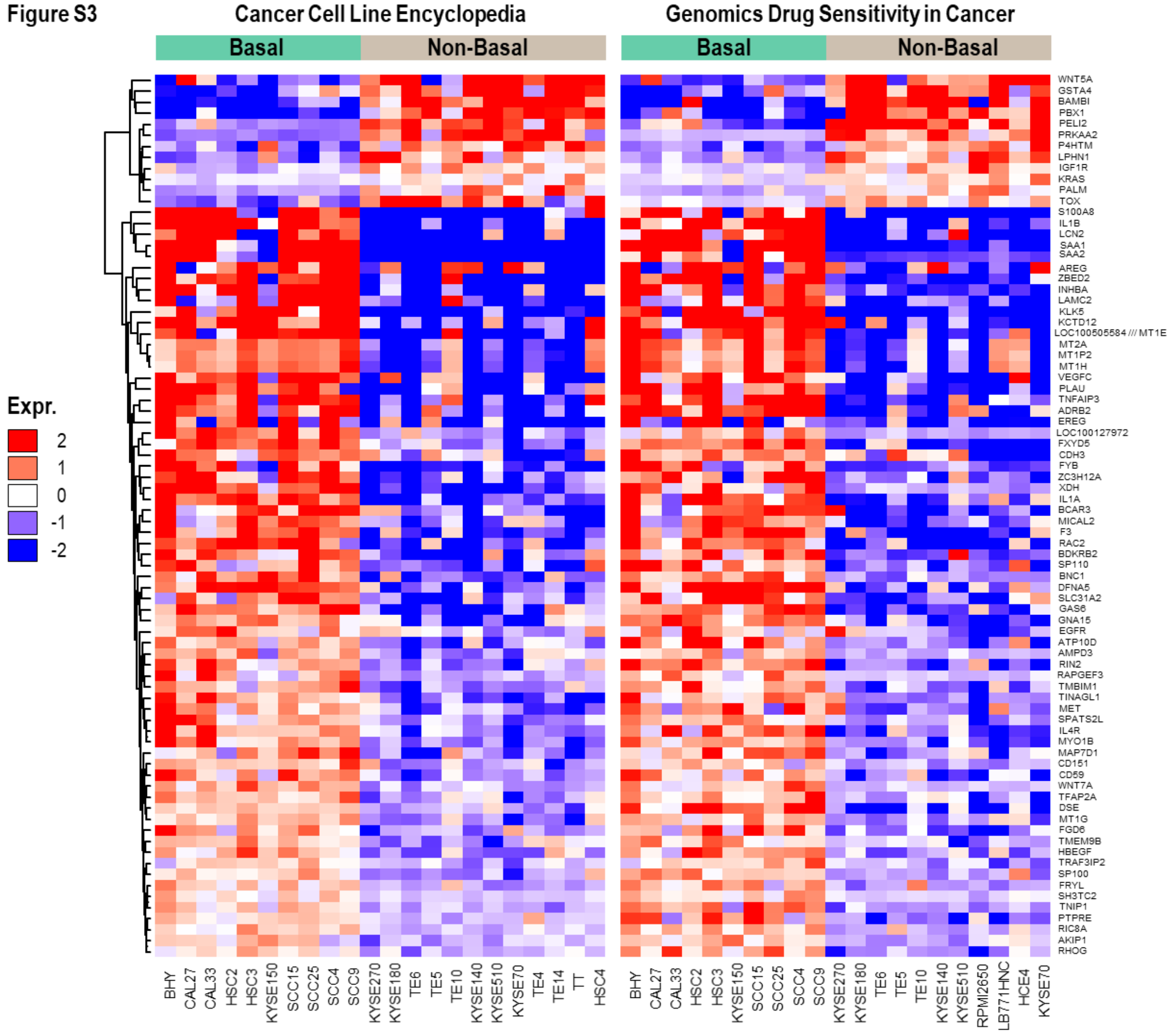

Figure S3. Heatmap of the differentially expressed genes between cell lines of the basal and non-basal subtypes in the CCLE [21] and GDSC [20] public datasets.

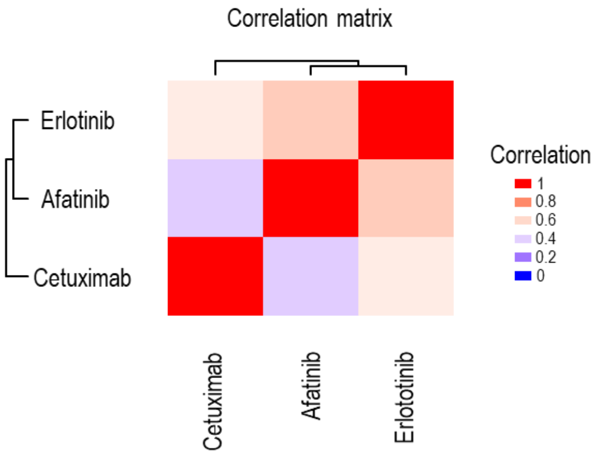

Figure S4. Correlation matrix of the AUC obtained upon treatment with Afatinib, Erlotinib and Cetuximab. A correlation of 1 is shown in red, and a correlation of 0 is shown in blue.

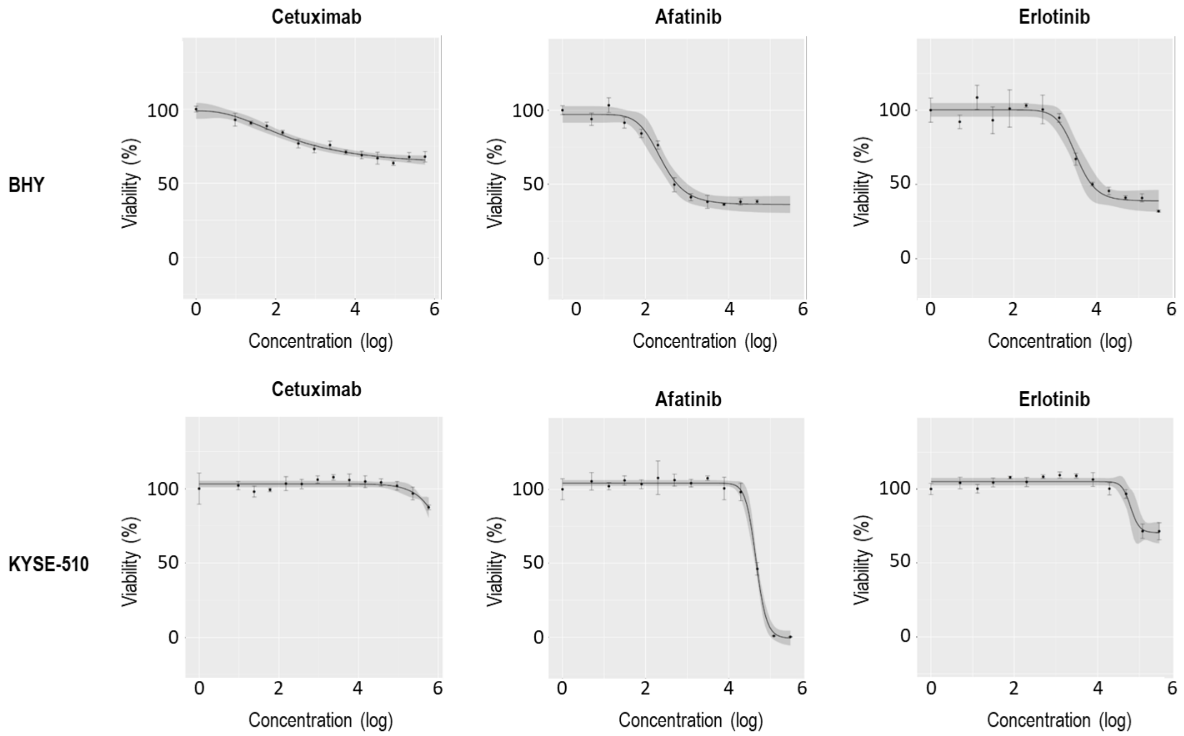

Figure S5. Cell viability dose-response curves obtained on BHY (upper panels) and KYSE-510 (lower panels) cells following 96 h of treatment with Cetuximab (left panels), Afatinib (middle panels) and Erlotinib (right panels).

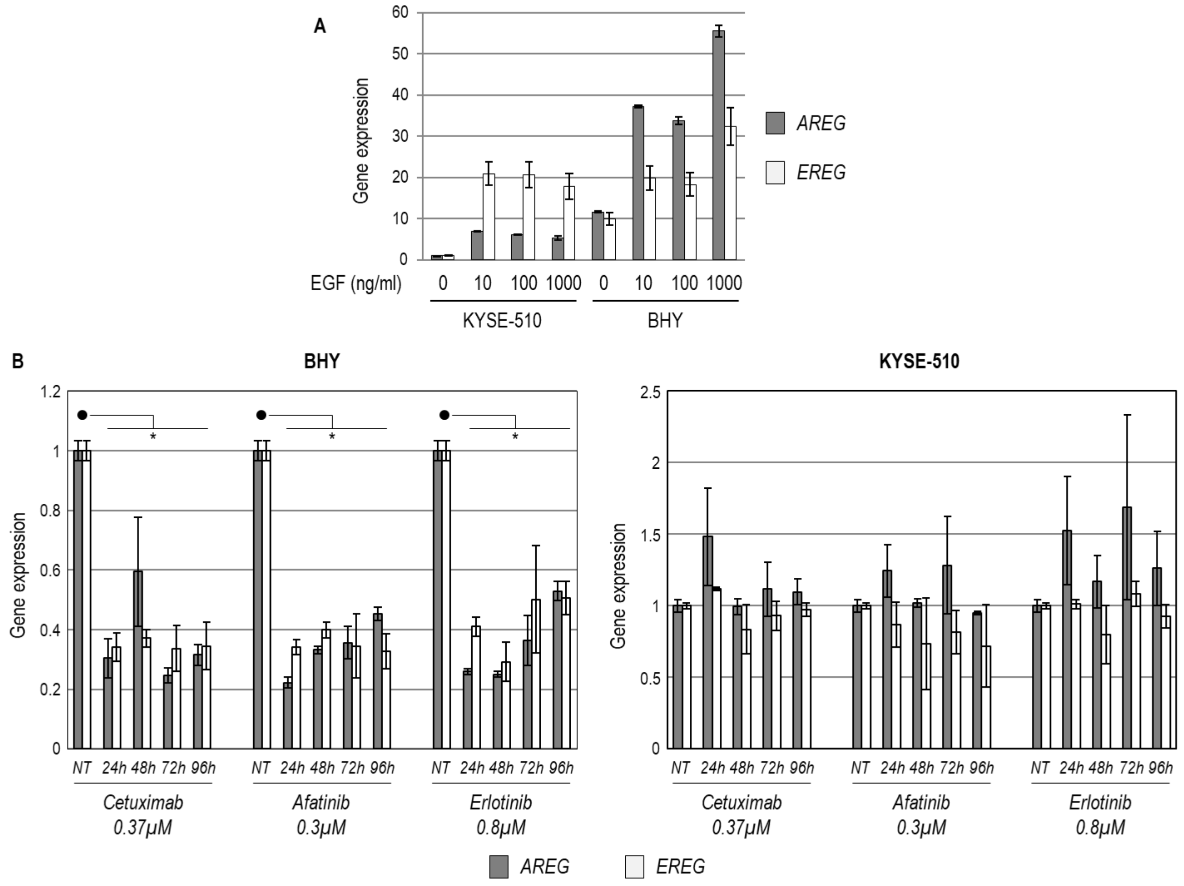

Figure S6. Analysis of *AREG* and *EREG* gene expression in BHY and KYSE-510 cell lines. (**A**) *AREG* and *EREG* gene expression analysis in BHY and KYSE-510 cells grown in the presence of 10 ng/mL,

100 ng/mL and 1000 ng/mL EGF for 24 h. Non-treated cells were used as a negative control and results were normalized to non-treated KYSE-510 cells. Mean expression levels and standard errors from two independent experiment are represented. (**B**) *AREG* and *EREG* gene expression analysis in BHY and KYSE-510 cells grown in the presence of 0.37 μM Cetuximab, 0.3 μM Afatinib and 0.8 μM Erlotinib for 24 h, 48 h, 72 h and 96 h. Results were normalized to non-treated cells used as negative controls in both cell lines. EGFR-blockade significantly (ANOVA and Student-Newman-Keuls test for pairwise comparison) downregulated the expression of *AREG* and *EREG* in BHY cells.

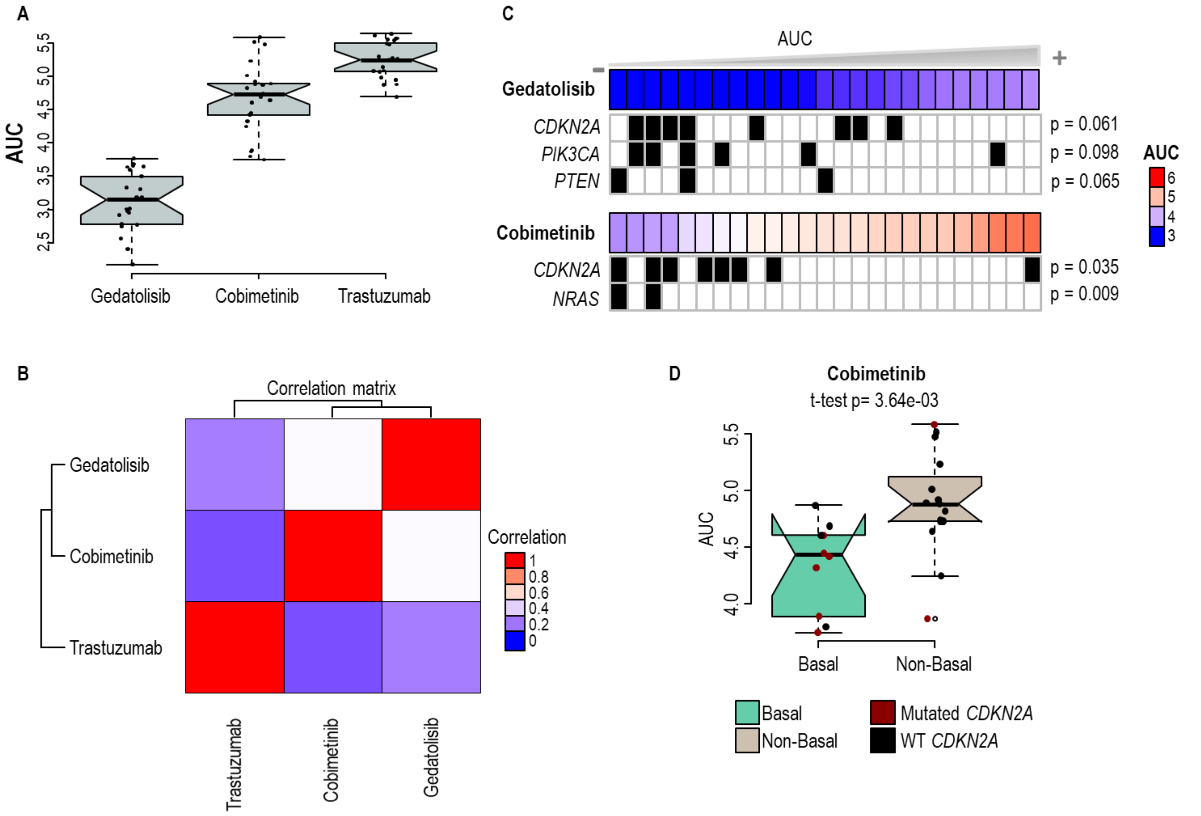

Figure S7. Analysis of the response of the 25 HNSCC cell lines to treatment with Gedatolisib, Trastuzumab and Cobimetinib. (**A**) Boxplot of the AUC for the 25 HNSCC cell lines treated with Gedatolisib, Trastuzumab and Cobimetinib. (**B**) Correlation matrix of the AUC obtained upon treatment with Gedatolisib, Trastuzumab and Cobimetinib. A correlation of 1 is shown in red, and a correlation of 0 is shown in blue. (**C**) Representation of clinically revelant mutations association with response to Gedatosilib and Cobimetinib. For each drug, cell lines are ordered by increasing AUC. *p*-values correspond to moderate *t*-test *p* values comparing AUC between mutated and WT cell lines. (**D**) Boxplot representation of the AUC obtained after Cobimetinib treatment of basal-like and non-basal HNSCC cell lines. The AUC were found to be significantly lower (*t*-test *p*-values are shown) in basal-like cells.

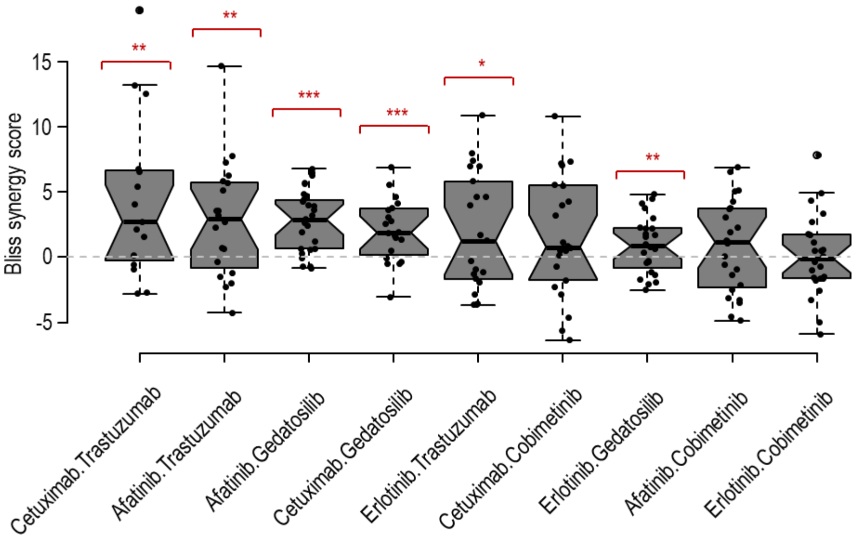

Figure S8. Boxplot of the Bliss synergy scores for the 25 cell lines treated with the nine drug combinations. *p* values correspond to moderate *t*-test *p* values comparing Bliss synergy score to 0: *: *p* value in [0.05, 0.1], **: *p* value in [0.001, 0.05] and ***: *p* value < 0.001. Red brackets indicate therapeutic combinations that were found to be synergistic on all cell lines.

© 2019 by the authors. Licensee MDPI, Basel, Switzerland. This article is an open access article distributed under the terms and conditions of the Creative Commons Attribution (CC BY) license (http://creativecommons.org/licenses/by/4.0/).
